# Determinants of success and sustainability of the WHO multimodal hand hygiene promotion campaign, Italy, 2007–2008 and 2014

**DOI:** 10.2807/1560-7917.ES.2017.22.23.30546

**Published:** 2017-06-08

**Authors:** Maria Luisa Moro, Filomena Morsillo, Simona Nascetti, Mita Parenti, Benedetta Allegranzi, Maria Grazia Pompa, Didier Pittet

**Affiliations:** 1 Agenzia Sanitaria e Sociale Regione Emilia-Romagna, Bologna, Italy; 2 Dipartimento di Sanità Pubblica, Area Igiene e Sanità Pubblica, Azienda USL, Bologna, Italy; 3 Azienda Ospedaliero-Universitaria di Bologna Policlinico S.Orsola-Malpighi, Bologna, Italy; 4 Infection Prevention and Control Global Unit, WHO Service Delivery and Safety, World Health Organization, Geneva, Switzerland; 5 Ufficio 5 - Rapporti con l'Unione Europea, il Consiglio d'Europa, l'OCSE, l'OMS, e le altre agenzie ONU ed Organizzazioni internazionali, Direzione generale della comunicazione e dei rapporti europei e internazionali, Ministero della Salute, Rome, Italy; 6 Infection Control Programme and WHO Collaborating Centre on Patient Safety, University of Geneva Hospitals and Faculty of Medicine, Geneva, Switzerland

**Keywords:** Hand hygiene, compliance, multimodal promotion strategy, national campaign, HAI, World Health Organization, sustainability

## Abstract

A national hand hygiene promotion campaign based on the World Health Organization (WHO) multimodal, Clean Care is Safer Care campaign was launched in Italy in 2007. One hundred seventy-five hospitals from 14 of 20 Italian regions participated. Data were collected using methods and tools provided by the WHO campaign, translated into Italian. Hand hygiene compliance, ward infrastructure, and healthcare workers’ knowledge and perception of healthcare-associated infections and hand hygiene were evaluated before and after campaign implementation. Compliance data from the 65 hospitals returning complete data for all implementation tools were analysed using a multilevel approach. Overall, hand hygiene compliance increased in the 65 hospitals from 40% to 63% (absolute increase: 23%, 95% confidence interval: 22–24%). A wide variation in hand hygiene compliance among wards was observed; inter-ward variability significantly decreased after campaign implementation and the level of perception was the only item associated with this. Long-term sustainability in 48 of these 65 hospitals was assessed in 2014 using the WHO Hand Hygiene Self-Assessment Framework tool. Of the 48 hospitals, 44 scored in the advanced/intermediate categories of hand hygiene implementation progress. The median hand hygiene compliance achieved at the end of the 2007–2008 campaign appeared to be sustained in 2014.

## Introduction

In recent years, increasing attention has been given to hand hygiene as a leading measure to prevent the spread of antimicrobial resistance and to reduce healthcare-associated infections (HAIs) [1]. Several studies offer convincing evidence that improved hand hygiene practices lead to a reduction of HAIs and/or transmission or colonisation by multidrug-resistant organisms (MDROs) [2].

In October 2005, the World Health Organization (WHO) launched Clean Care is Safer Care, a global hand hygiene campaign [3]. Aimed to reduce HAIs it focused on implementing new hand hygiene recommendations [4] through a multimodal hand hygiene promotion campaign [5]. At that time, the Italian Minister of Health signed a statement to show its commitment to reducing HAIs [6]. Consequently, a national hand hygiene campaign based on the materials provided by WHO was launched in November 2006. The campaign was organised by the national coordinating centre for HAIs (Agenzia Sanitaria e Sociale Regionale Emilia-Romagna) and funded by the National Centre for Disease Control (Centro Nazionale per la Prevenzione e il Controllo delle Malattie). The multimodal national campaign was conducted in 2007–2008 and not repeated in the following years.

This study reports the campaign’s effect on hand hygiene compliance immediately after implementation, and identifies factors associated with the observed improvement at the individual level and at the ward level. It also reports on the level of hand hygiene compliance 7 years later.

## Methods

### Implementing WHO’s Clean Care is Safer Care campaign

In November 2006, Italian public hospitals were invited by regional coordinators to implement the WHO’s Clean Care is Safer Care hand hygiene promotion campaign. Hospitals were asked to have at least one or two wards with at least one hand washing basin for every 10 beds participate, with intensive care units (ICUs), surgical wards or onco-haematology/transplant wards being most preferred.

The Italian campaign was based entirely on the WHO’s [3], and involved all WHO documents and tools [7] being translated into Italian and then made available on the Ministry of Health website [8]. The campaign tools were focused on the following five elements: (i) system change, including access to alcohol-based hand rub (ABHR); (ii) healthcare workers’ (HCWs) training and education; (iii) monitoring and feedback on practices; (iv) visual reminders in the workplace; and (v) institutional patient-safety climate [5,9].

Implementation of the campaign occurred from November 2006 onwards and in the following four stages: (i) preparedness (3 months on average); (ii) baseline evaluation (2.5 months on average); (iii) intervention (3 months on average); and (iv) follow-up evaluation (2.5 months on average) [5].

### Evaluating short-term impact of the campaign

The follow-up evaluation (implementation stage 4 as per above) was carried out from March 2007 to October 2008. Only hospitals/wards with data available from both the baseline evaluation and follow-up evaluation phases were included in the analysis.

Participating hospitals were requested to send all data collected via four questionnaires, provided by the WHO campaign [5,7] and translated into Italian [8], for facility situation (at baseline only), ward infrastructure, hand hygiene knowledge (anonymous) and hand hygiene perception (anonymous), as well as observations about hand hygiene compliance to the Agenzia Sanitaria e Sociale Regionale Emilia-Romagna. Hand hygiene perception explored HCW’s perceptions about their own hand hygiene compliance, the compliance of other HCWs, the impact of HAIs, the importance of hand hygiene as a preventive measure to reduce HAIs and the effectiveness of the different elements of a multimodal strategy. A total score for each of the four questionnaire areas [5,7] was calculated as the ratio of the response score to the maximum expected score (i.e. sum of the all items in the questionnaire/(overall maximum expected score × number of non-missing items)). Thus, each of the areas received scores ranging from 0 to 1.

Data on hand hygiene compliance was collected via a trained, unobtrusive observer who, during 20-minute sessions, openly observed staff and recorded the total number of hand hygiene opportunities and actions, either hand washing or hand rubbing [5,7]. An opportunity for hand hygiene was defined as the occurrence of any indication for hand hygiene according to the WHO ‘My 5 Moments’ approach [7,9,10]. Each ward had to record at least 200 opportunities; otherwise they were considered to have incomplete data and were excluded from the analysis [11].

### Determining long-term sustainability of the 2007–2008 campaign in 2014

The long-term sustainability of hand hygiene behaviour change was assessed 7 years after the conclusion of the national campaign. In 2014, the 65 hospitals included in the follow-up evaluation of the 2007–2008 campaign were invited to complete the Hand Hygiene Self-Assessment Framework (HHSAF). This tool is part of the WHO Clean Care is Safer Care kit [12,13], but was not a part of the tools used in the baseline and follow-up evaluations. The questionnaire comprises 27 items grouped into five sections reflecting the five elements of the multimodal campaign (e.g. system change; training and education; evaluation and feedback; reminders in the workplace; and institutional safety climate). Each component section is scored out of 100 points (total maximum score: 500), and based on this, responding healthcare facilities were classified as inadequate (≤ 125 points), basic (126–250), intermediate (251–375) or advanced (> 375) [12,13]. Notably, the HHSAF asked hospitals to report on the level of hand hygiene compliance obtained through direct observation.

### Statistical analysis

The following statistical methods were used to analyse the impact of the campaign.

Pearson’s chi-squared test and McNemar test were used where appropriate to investigate the difference between proportions.

For facility situation, ward infrastructure, hand hygiene knowledge and hand hygiene perception, the Cronbach’s alpha coefficient was used to estimate the reliability of sets of questionnaire items before calculating the scores (alpha values higher than 70% were considered acceptable) and the internal consistency between different items [14]. A non-parametric K-sample test on the equality of medians was used to test the differences among scores.

Compliance data were analysed using a multilevel approach [15,16] with hand hygiene opportunities as first level and ward characteristics as second level. The following first level covariates were used: professional category, hand hygiene indication and study phase (baseline and follow-up). Second level covariates were ward specialty, type of facility, the score related to the facility situation before the intervention, the ward structure score for hand hygiene, and HCWs’ knowledge and perception score in each ward before and after the campaign implementation. The number of observed opportunities per hour of observation [17,18] and the average hand hygiene compliance at baseline were used in the model evaluating the impact of the campaign.

A logistic multilevel regression model at mixed effects with binomial distribution and logit link was used to explore the effect of hand hygiene indications and professional categories taking different ward characteristics into account [19]. Two random intercept models were fitted, separately for baseline and follow-up phases, to investigate whether there was significant clustering within wards in relation to hand hygiene compliance, to which extent the variance among wards was explained by ward opportunities mix, and whether specific ward characteristics were associated with compliance variance among wards.

The intra-class correlation (ICC) was used to measure the proportion of the overall variance in hand hygiene compliance explained by the clustering variable. Compliance at baseline in each ward was used to correct the compliance variance estimate. The proportional change in variance (PVC) was used to measure the reduction of variance compared with the empty model or to the previous model. A bivariate analysis was used, overall and also separately for the three types of ward (surgical, intensive care, and medical/other wards) to investigate whether the relative change in hand hygiene compliance was statistically associated with the relative change in ward infrastructure, hand hygiene knowledge and hand hygiene perception questionnaire scores at ward level at follow-up. The relative change in compliance was defined as the absolute difference between two overall measurements (at baseline and follow-up) compared with the baseline measurement in each ward. We applied a linear regression model with bootstrap estimation.

Statistical analyses were performed using STATA/IC 11.1.

## Results

Fourteen of 20 regions in Italy agreed to actively participate in the campaign, leading to a total involvement of 175 hospitals comprised of 285 wards. Of the 175 hospitals, 65 returned complete data (37.1%). The variation in participation across regions was not explained by any particular reason. Of the 285 wards, 200 were excluded; 190 (95%) because they were unable to perform all of the requested hand hygiene observations and 10 (5%) because not all the questionnaires were sent back. This left 85 wards (29.8%) remaining for the analysis, which were included. The 65 hospitals included in the analysis were similar to the 110 excluded hospitals in terms of public ownership (83% vs 80%) and infection control score before the campaign (40% vs 39%). The 85 wards included in the analysis were more frequently intensive care units (ICUs) compared with the 172 excluded wards (56% vs 45%, chi-square 3.1, p value = 0.038).

In terms of observed compliance, a total of 18,045 opportunities for hand hygiene were recorded at baseline and 17,577 at follow-up; the number of observation sessions was 1,643 at baseline and 1,403 at follow-up with a median duration of 20 minutes for both baseline and follow-up (IQR: 15 and 10, respectively). The median number of opportunities observed per hour was 20 for baseline and 24 for follow-up (IQR: 24 and 21, respectively). The distribution of observed opportunities was similar during both phases (Table 1).

**Table 1:** Compliance with hand hygiene at baseline and follow-up across professional categories, type of indication and ward, national campaign, Italy, 2007–2008

	Baseline		Follow-up			
	Opportunities (n)	Compliance (%)	Opportunities (n)	Compliance (%)	Absolute difference in compliance (%)	95% CI
**Overall**	18,045	40	17,577	63	+ 23	22–24
**Professional category**						
Nurses	11,732	42	11,506	67	+ 25	24–26
Medical doctors	3,849	39	3,693	55	+ 16	14–18
Auxiliary	1,960	33	2,114	61	+ 28	25–31
Other	504	28	264	46	+ 18	11–25
**Hand hygiene indication**						
Before patient contact	5,538	33	5,494	59	+ 26	24–28
Before aseptic task	2,109	45	2,008	64	+ 19	16–22
After contact with patient surroundings	3,602	25	3,141	50	+ 25	23–27
After patient contact	5,117	50	5,070	71	+ 21	19–23
After body fluid exposure risk	1,679	55	1,864	75	+ 19	16–22
**Type of ward**						
Surgical	4,762	31	4,735	56	+ 25	23–27
Intensive care	10,618	42	10,076	65	+ 23	22–24
Medical and other wards	2,665	45	2,766	67	+ 22	20–25
**Type of hospital**						
Private	599	25	589	45	+ 20	15–26
Public	15,511	40	15,190	63	+ 23	22–24
Research/teaching	1,935	41	1,798	71	+ 30	27–33

CI: confidence interval.

Of the 65 hospitals included in the 2007–2008 follow-up evaluation, 48 participated in the 2014 HHSAF survey to assess the level of implementation of hand hygiene over time: 40 of 56 public hospitals, six of seven teaching hospitals, and two of two private hospitals.

### Impact of campaign, 2007–2008

At baseline, the facility situation median score (measured at baseline only) was high (0.77) and showed small inter-hospital variation (IQR: 0.143), but the ward infrastructure median score was low (0.50) with considerable inter-ward variation (IQR: 0.50). At follow-up, scores arising from the three baseline/follow-up questionnaire areas increased significantly: the ward infrastructure median score increased to from 0.50 to 0.83 (p value < 0.0001), the hand hygiene knowledge median score increased from 0.53 to 0.68 (p value < 0.0001) and the hand hygiene perception median score increased from to 0.69 to 0.77 (p value < 0.0001).

Overall, hand hygiene compliance increased from 40% to 63% (absolute increase: 23%, 95% confidence interval (CI): 22–24%). Compliance significantly increased across all professional categories, types of hand hygiene indications, types of wards and types of hospitals, with the extent of compliance increase being greatest for those areas that showed low compliance before intervention (Table 1).

At baseline, hand hygiene compliance was significantly associated with the type of professional category, the type of hand hygiene indication, the type of ward speciality, and the ward structure for hand hygiene score. Compliance was highest for the professional category of nurses (p < 0.0001), for the indication of ‘after body fluid exposure risk’ (p value < 0.0001), and for medical wards (p value < 0.05) (Table 2). At follow-up, compliance was significantly associated with the type of professional category, the type of hand hygiene indication and the perception score (Table 2).

**Table 2:** Factors associated with hand hygiene compliance at baseline and follow-up, national campaign, Italy, 2007–2008

	Baseline				Follow-up			
	OR	95% CI	p value		OR	95% CI	p value	
**Hand hygiene indication**								
After body fluid exposure risk	Ref.	NA	NA		Ref.	NA	NA	
After patient contact	0.75	0.65–0.85	< 0.0001		0.78	0.68–0.89	< 0.0001	
After contact with patient surroundings	0.28	0.24–0.32	< 0.0001		0.32	0.28–0.37	< 0.0001	
Before patient contact	0.32	0.28–0.37	0.004		0.45	0.39–0.52	< 0.0001	
Before aseptic task	0.59	0.50–0.68	< 0.0001		0.54	0.46–0.64	< 0.0001	
**Professional category**								
Nurses	Ref.	NA	NA		Ref.	NA	NA	
Medical doctors	0.74	0.68–0.81	< 0.0001		0.54	0.49–0.59	< 0.0001	
Auxiliary	0.69	0.61–0.79	< 0.0001		0.81	0.72–0.92	< 0.0001	
Other professionals	0.48	0.38–0.61	< 0.0001		0.31	0.23–0.42	< 0.0001	
**Type of hospital**								
Research/teaching	Ref.	NA	NA		Ref.	NA	NA	
Private	1.39	0.26–2.86	0.643		1.00	0.24–2.41	0.995	
Public	2.00	0.50–3.56	0.080		1.10	0.10–3.04	0.801	
**Facility situation score greater than the median^a^**	1.12	0.09–3.03	0.645		1.35	0.13–3.49	0.211	
**Type of ward**								
Medical and other wards	Ref.	NA	NA		Ref.	NA	NA	
Surgical ward	0.48	0.35–0.85	0.047		0.69	0.66–1.92	0.296	
ICU	1.08	0.54–1.85	0.818		0.91	0.78–3.13	0.770	
**Ward infrastructure for hand hygiene score greater than the median^a^**	2.05	1.50–7.06	0.015	1.50		0.70–12.40	0.093	
**Knowledge score greater than the median^a^**	1.42	0.19–5.65	0.144	1.28		0.24–6.76	0.313	
**Perception score greater than the median^a^**	0.88	0.07–1.35	0.637	1.75		1.50–32.50	0.022	
**Measures of hand hygiene compliance variability**								
Ward level variance^b^	0.92	0.69–1.37	NA	0.85		0.63–1.27		NA
Change in variance^c^	-22%	NA	NA	-30%		NA		NA
Intra-class correlation^d^	22%	NA	NA	20%		NA		NA

CI: confidence interval; ICU: intensive care unit; OR: odds ratio.

^a^ Dummy variable yes vs no.

^b^ Standard Error (SE) in the empty model is 1.182 (0.203) and 1.213 (0.216), baseline and follow-up respectively.

^c^ Proportional reduction change in variance (PVC).

^d^ Intra-class correlation coefficient (ICC) in the empty model is 26% and 29%, baseline and follow-up respectively.

Notably, a wide variation in hand hygiene compliance among wards was observed at baseline and follow-up. The variance was significant in the empty models both before and after the intervention, and both the before and after ICCs were high (Table 2). At baseline, inter-ward variability of hand hygiene compliance after adjusting for major confounders was seen, with ICUs and medical/other wards having significantly higher compliance than surgical wards (p value = 0.047). This inter-ward variability decreased after campaign implementation, with perception being the only factor significantly associated with this (p value = 0.022). In ICUs and surgical wards, perception was strongly associated with increased hand hygiene compliance at follow-up. Correlating the relative change in overall hand hygiene compliance to the changes in the hand hygiene perception, hand hygiene knowledge and ward infrastructure scores showed that only hand hygiene perception scores were significantly associated with the change (beta-coefficient: 0.343, p value < 0.0001).

### Long-term sustainability of campaign, 2014

Of the 48 hospitals that completed the HHSAF, the median HHSAF score was 345 (Interquartile range (IQR): 83.7) and 44 were in the intermediate or advanced levels of hand hygiene implementation progress. Overall, the highest component score was for system change (median 100.0, IQR: 20), while the lowest was for institutional safety climate (median 50, IQR: 35). All 12 hospitals that reached the advanced level completed the leadership section; their median leadership score was 14.0 (IQR: 4).

Table 3 describes the 48 hospitals using data collected during the baseline and follow-up evaluations of the 2007–2008 campaign, and data collected during the 2014 HHSAF survey.

**Table 3:** Survey results of hospitals participating in the 2007–2008 national campaign according to 2014 hand hygiene implementation level, Italy, 2014 (n = 48 hospitals)

	Implementation level in 2014			
	Advanced (n = 12)		Intermediate/basic (n = 36)	
***2007–2008 campaign***	**Median**	**Interquartile range**	**Median**	**Interquartile range**
Hand hygiene compliance (observed/expected)				
at baseline	0.52	0.22^a^	0.37	0.28^a^
at follow-up	0.74	0.21	0.63	0.28
Ward infrastructure for hand hygiene score (questionnaire)				
at baseline	0.67	0.25^a^	0.33	0.50^a^
at follow-up	0.84	0	0.83	0.17
Knowledge score (questionnaire)				
at baseline	0.53	0.07	0.53	0.07
at follow-up	0.75	0.24	0.74	0.26
Perception score (questionnaire)				
at baseline	0.77	0.11^a^	0.69	0.06^a^
at follow-up	0.84	0.08	0.77	0.07
***2014 survey***	**Median score/max achievable score**	**Interquartile range**	**Median score/max achievable score**	**Interquartile range**
HHSAF	450/500	60	322.5/500	77.5
Components scores of HHSAF				
System change	100/100	0	90/100	20
Training and education	100/100	10	65/100	10
Evaluation and feedback	80/100	10	55/100	35
Reminders in the workplace	90/100	20	65/100	12
Institutional safety climate for hand hygiene	90/100	30	40/100	20
Selected items of HHSAF				
Training and education^b^	40/40	0	20/40	20
Evaluation and feedback: hand hygiene compliance^c^	25/30	6	15/30	15
Institutional safety climate for hand hygiene^d^	20/20	0	15/20	11.2
Institutional safety climate for hand hygiene^e^	10/10	10	0/10	0
Institutional safety climate for hand hygiene^f^	5/5	0	0/10	5
Institutional safety climate for hand hygiene^g^	10/10	2.5	0/10	0

HHSAF: Hand Hygiene Self-Assessment Framework; IQR: interquartile range.

^a^ Advanced vs intermediate/basic: p value < 0.05.

^b^ Item 2.1a, 2.1b: Mandatory training for all professional categories at commencement of employment, then ongoing regular training (at least annually).

^c^ Overall hand hygiene compliance rate according to the WHO Hand Hygiene Observation tool (or similar technique): 25 points corresponds to compliance equal to 71–80%; 15 points corresponds to compliance of 51–60%.

^d^ Visible commitment to support hand hygiene improvement by the Chief Executive Officer, the Medical Director, the Director of Nursing.

^e^ A clear plan for the promotion of hand hygiene throughout the entire facility for the annual global campaign on 5 May, Save Lives: Clean Your Hands.

^f^ Patients informed about the importance of hand hygiene (e.g. via a leaflet).

^g^ A formalised programme of patient engagement.

Facilities classified as advanced via the HHSAF questionnaire in 2014 were already better performers when the 2007–2008 campaign was initiated: at baseline in 2007–2008, the median scores for hand hygiene compliance (0.52 vs 0.37, chi-squared test 4.28, p value = 0.0384), ward infrastructure (0.67 vs 0.33, chi-squared test 4.17, p value = 0.0411), and hand hygiene perception (0.77 vs 0.69, chi-squared test 7.47, p-value = 0.0063) were significantly higher for facilities classified as advanced compared with those classified as intermediate or basic in 2014. Observed changes between the baseline and follow-up evaluations were comparable between advanced and intermediate/basic facilities with the exception of ward infrastructure for hand hygiene: this improved more among facilities classified as intermediate or basic in 2014 (0.50 median absolute change, IQR: 0.50 vs 0.17 median absolute change, IQR: 0.17, chi-squared test 10.94, p value = 0.0009). In 2014, the median hand hygiene compliance achieved at the end of the 2007–2008 campaign appeared to be sustained. For the 12 hospitals classified as advanced in 2014, the median reported hand hygiene compliance score was 25 points (corresponding to 71–80% compliance) in 2014 while the median observed hand hygiene compliance at follow-up was 74% in 2007–2008. For the 36 intermediate/basic hospitals, the median 2014-reported compliance was 15 points (corresponding to 51–60% compliance) while the median 2007–2008 observed compliance at follow-up was 63% (Table 3).

## Discussion

Implementation of a multimodal promotion campaign in 65 hospitals at the national level in Italy led to significant hand hygiene compliance improvement across all types of wards and professional categories.

The inclusion of several hospitals and wards across Italy allowed us to explore factors explaining the variability in hand hygiene compliance among different wards, both before and after campaign implementation. Consistent with previous reports [4], compliance varied across professional categories and types of hand hygiene indications. Nurses started with and achieved the highest level of hand hygiene compliance, consistent with a systematic review of 96 studies that showed median compliance rates 16% lower among physicians compared with nurses [20]. Compliance was highest with the indication ‘after contact with body fluids’ both at baseline and follow-up. This was consistent with several studies that have demonstrated that hand hygiene action is more frequently performed after contact with body fluids or after patient contact than before, possibly suggestive of self-protection against harmful organisms [4,21].

Compliance increase was indeed accompanied by a parallel improvement in all factors shown to influence hand hygiene behaviour: availability of hand hygiene products within the ward, knowledge of hand hygiene principles, perception of the importance of hand hygiene and of multimodal actions to improve hand hygiene. This is consistent with that reported in other campaigns [9].

The results of the study show that perception characterises the variability in hand hygiene compliance across wards following intervention, and confirms that improving perception of HAIs and hand hygiene improve hand hygiene behaviour.

Others have also found that hand hygiene is influenced by the perceived behaviour of other healthcare professionals [22] and that education methods to enhance perception are requisites for success [18]. In our campaign, several innovative methods were used to promote knowledge and perceptions, such as videos of real-life situations, experiential learning and participatory sessions. Given that others have found that using innovative methods is a key factor for success [21], it is anticipated that the innovative methods used to promote improved knowledge and perception in this study may have been effective at addressing existing behavioural barriers to hand hygiene, especially in settings where compliance was low, such as surgical wards.

Understanding which factors may be important to reducing the variability across wards is of paramount importance to achieving a uniform high level of hand hygiene compliance. Saint et al. found a significant variability of hand hygiene compliance across five wards in Tuscany: the highest performing ward was characterised by the commitment of its physician leaders to hand hygiene improvement and the early adoption of ABHR [23]. Our results confirm both the feasibility and the effectiveness of a large scale implementation of a multimodal hand hygiene promotion campaign, thereby supporting the systematic review and network meta-analysis recently published by Luangasanatip et al [24].

Few studies have reported on long-term hand hygiene compliance [24], especially after a one-off national campaign. The long-term results of the HHSAF survey conducted in 2014 are very encouraging: 7 years after the end of the campaign and without any campaigning activity at the national level thereafter, hospitals that implemented the campaign were still actively promoting hand hygiene. Of the 48 hospitals completing the HHSAF survey, 44 were classified as intermediate or advanced in terms of hand hygiene implementation progress. This compares with 94% (122/129) in the most recent similar survey conducted in the United States using the same tool [13].

All 48 hospitals were still implementing the core components of the WHO campaign even though the campaign itself was not repeated: training scored 40/40 in advanced hospitals vs 20/40 in the other hospitals; hand hygiene direct observation was claimed to still be in place and 71–80% compliance was reported by advanced hospitals vs 51–60% in the others; visible commitment to hand hygiene was assured by top managers scoring 20/20 in advanced hospitals vs 15/20 in the others. Institutions classified as advanced in 2014 were already more prone to hand hygiene promotion at baseline in 2007–2008.

Our study has limitations. First, while we explored the effect of both individual variables, such as professional category and hand hygiene indications, as well as hospital and ward characteristics on compliance, we could not consider additional factors such as the potential positive role of opinion leaders or early adopters. Such investigations are extremely difficult to conduct on a large scale and should be considered as next steps. Second, since hospital participation was voluntary, those participating were possibly more inclined to improve than others. The median facility situation score showing 0.77 at baseline was indeed high with small inter-hospital variability, while Italian national data show significant variation in the development of infection control organisations and initiatives by region and type of hospital [25]. Moreover, we only received full 2007–2008 follow-up data from a subsample of the 175 participating hospitals (37% (n = 65)) mainly due to the unavailability of all the requested data (knowledge, perception, and ward questionnaires and/or hand hygiene observations). Based on the information available, the 65 participating hospitals were similar to the excluded ones in terms of public ownership and infection control activities in place before the campaign. One difference was that the participating hospitals have more ICUs, but the possible effect on the results was minimal. Third, the so called ‘Hawthorne effect may have occurred [26]; however, its overall impact is difficult to quantify when the direct observation of practices is conducted and it cannot explain the observed uniform improvement shown across all sites, wards and professional categories in 2007–2008 or the observed sustainability 7 years later. Fourth, this study is limited by the absence of data on patient outcomes, which it was not designed to monitor. Given the high number of enrolled wards not performing HAI surveillance, implementing infection surveillance to evaluate the impact of the campaign on patient outcomes for the duration of the study or using available surveillance data from a proportion of wards only was considered unfeasible and potentially inaccurate. However, a large number of other studies have explored the link between hand hygiene and infection rates [1] and investigators continue to add positive evidence [27-30].

In conclusion, the national campaign using a translated version of the WHO Clean Care is Safer Care materials was effective in improving hand hygiene compliance across 65 hospitals in Italy in 2007–2008: increased perception of HAIs and hand hygiene was an important driver for improvement. The campaign, which was not repeated in following years, seems to have contributed to a good level of hand hygiene 7 years later in 48 of these hospitals.
